# Associations of bone mineral density with sex hormone-binding globulin (SHBG) and testosterone in middle-aged Saudi men: a cross-sectional study

**DOI:** 10.3389/fendo.2023.1230279

**Published:** 2023-11-24

**Authors:** Sobhy M. Yakout, Malak Nawaz Khan Khattak, Nasser M. Al-Daghri, Abeer A. Al-Masri, Mohamed A. Elsaid

**Affiliations:** ^1^ Chair for Biomarkers of Chronic Diseases, Department of Biochemistry, College of Science, King Saud University, Riyadh, Saudi Arabia; ^2^ Department of Physiology, College Medicine, King Saud University, Riyadh, Saudi Arabia

**Keywords:** SHBG, testosterone, fAI, BMD, osteopenia

## Abstract

**Objective:**

The present cross-sectional study examined the association between circulating levels of sex hormone-binding globulin (SHBG) and testosterone with bone mineral density (BMD) in middle-aged Arab men.

**Methods:**

Clinical data of 103 middle-aged Saudi men (mean age 60.7±7.2) were extracted from the Osteoporosis Registry of the Chair for Biomarkers of Chronic Diseases, King Saud University in Riyadh, Saudi Arabia. Participants were categorized according to the presence of osteopenia (T-score -1.0 to -2.5) (N=47) and controls (N=56). Data collected included demographics and anthropometrics as well as levels of sex hormone-binding globulin (SHBG), testosterone and follicle-stimulating hormone (FSH) which were measured using commercially available assays. Free androgen index (FAI) was calculated.

**Results:**

Those with osteopenia had significantly lower levels of FAI (p<0.05), and higher levels of SHBG (p<0.004) and FSH (p<0.005). In the osteopenia group, SHBG was positively correlated with age (r=0.33, p<0.05), while it was inversely correlated with BMD spine (r = -0.39, p<0.05) and T-score femur (r= -0.35, p<0.05) in the same group. Furthermore, testosterone was inversely correlated with BMI in the osteopenia group (r= -0.33, p<0.05) while FAI was positively correlated with T-score femur (r = 0.36, p<0.05) as well as in all participants (r= 0.24, p<0.05). Among controls, FAI had an inverse correlation with FSH (r= -0.28, p<0.05) and over-all (r= -0.22, p<0.05).

**Conclusion:**

In summary, the associations elicited suggest that circulating levels of SHBG and FAI may be against age-related bone loss in middle-aged men.

## 1 Introduction

The aging population is growing quickly around the world, making age-related diseases such as osteoporosis a major public concern (1). Osteopenia, often regarded as a precursor to osteoporosis, can go unnoticed until it progresses, leading to an increased fracture risk in elderly patients, psychological and physical harm (2). This can also be a financial burden on families and society. Prevention is the best way to manage osteoporosis, so it is essential to pay close attention to those at risk, e.g., those with osteopenia and take early action for prevention and treatment.

At the start of the 21st century, reports showed that the prevalence of osteoporosis in Saudi Arabia was more severe than in other parts of the world (3). Early findings indicated that as many as 48% of people had osteopenia (4–6). Gouhar et al. observed that 29.7% of people over the age of 60 with osteoporosis were in this age group (7). Al Quaiz et al. also used DXA to check the bones of 362 healthy women and found that 58.6% of them had low bone density, even though most of them were between 40 and 50 years old (8).

Despite the extensive research conducted on osteoporosis in Saudi Arabia, there is a notable gap when it comes to studying patients specifically diagnosed with osteopenia. Consequently, there is a crucial need to identify and address the occurrence of osteopenia in Saudi men, with the ultimate aim of enhancing targeted interventions for both prevention and management of osteoporosis. Addressing this gap is essential to devise effective strategies for the prevention and management of osteoporosis in this demographic.

In men, the deficit of sex hormones is linked to diminished bone strength and heightened fracture risk, with the quality of the bone being a determining factor (9, 10). Bone mineral density (BMD) is affected by both genetic and environmental factors and is closely linked to osteoporosis and bone fractures. Numerous studies have investigated into the relationship between adult male sex hormone levels and BMD, and a prevalent finding has been an inverse correlation between Sex Hormone-Binding Globulin (SHBG) levels and BMD (11–13). SHBG, a protein binding firmly to sex steroids in the bloodstream, has its levels influenced by a plethora of factors (14). While certain studies propose that elevated SHBG levels in men could elevate osteoporosis and fracture risks, even after accounting for sex hormone levels (15–18), other research has found no significant impact of SHBG on BMD after considering other factors (19).

Despite advances in understanding, there remains an unsettling gap in patient care. Many men at risk are not routinely screened to ascertain fracture probability nor are they educated about fracture prevention or prevention and treatment of osteoporosis (20). Globally, questions arise regarding the frequency of BMD and DXA screenings in men. How often are men, especially those at risk, screened to ascertain their fracture probability? What guidelines dictate the criteria for these screenings? Particularly in Saudi Arabia, understanding SHBG’s role in bone health among aging males remains underexplored. This study aims to bridge this gap by investigating the correlations between SHBG levels, testosterone and other sex hormones with BMD in middle-aged Saudi men, thereby providing valuable insights to enhance osteoporosis prevention and management strategies.

## 2 Materials and methods

### 2.1 Study subjects

This study included a total of 103 Saudi men from the Osteoporosis Registry of the Chair for Biomarkers of Chronic Diseases (CBCD) at King Saud University in Riyadh, Saudi Arabia (21). In brief, the osteoporosis registry is a database of adult patients aged 55 years and above, from all over Riyadh tertiary hospitals who underwent DXA scan and provided blood samples and consent to be included in the registry for research purposes. Mean value of Bone mineral density (BMD) measurements were taken at the lumbar spine, left hip, and right hip using DXA (Hologic QDR 2000 Inc., Woltham, MA, USA). A patient with a T-score between -1 to -2.5 (mean value standard deviations, SD) is classified as having osteopenia (22). The exclusion criteria included men with pre-existing bone diseases other than low BMD or under medications that can affect BMD for at least 6 months. The participants were categorized into two groups: 47 individuals with osteopenia and 56 individuals with normal BMD. Osteopenia was defined as having a T-score between −2.5 and −1.0, while those with a T-score above −1.0 were considered to have normal BMD. Various anthropometric measurements were recorded, including height, weight, waist and hip circumferences, as well as systolic and diastolic blood pressure. Body mass index (BMI) was calculated by dividing weight (kg) by height (m^2^).

**Figure 1:**
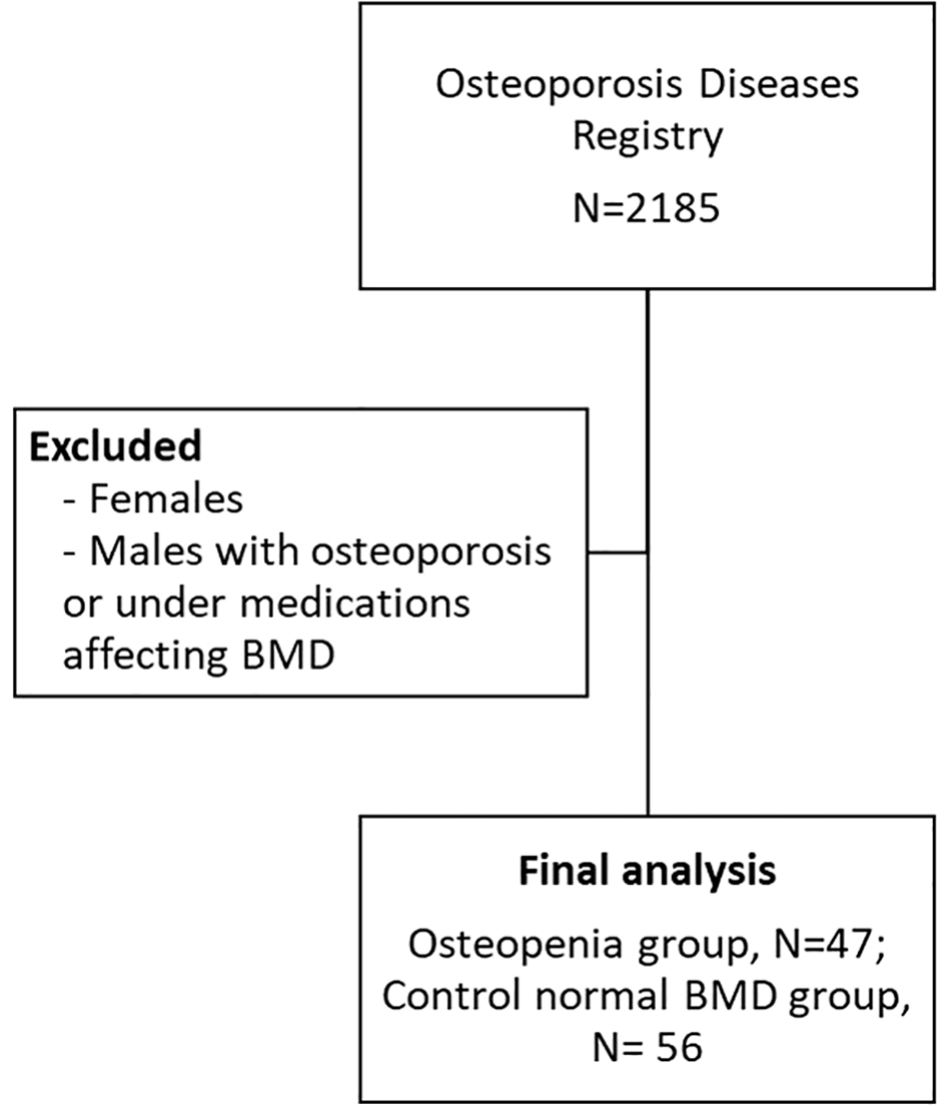
Flowchart of participants.

Certain exclusion criteria were applied in this study. Men with pre-existing bone diseases other than low BMD, those using or under medications that can affect BMD for at least 6 months, individuals with a history of treatment with pulsed electromagnetic fields (PEMFs) were excluded. The study was conducted in accordance with the Declaration of Helsinki and received approval from the Ethics Committee of the College of Science, King Saud University, Riyadh, Kingdom of Saudi Arabia (Approval# 8/25/454266, 30 September 2013). **Figure 1** shows the flowchart of participants.

### 2.2 Biochemical analysis

Blood samples were collected from participants after an overnight fasting period. The measurement of SHBG was performed using an electrochemiluminescence immunoassay with the Roche Cobas-e411 kit (Roche Diagnostics, Mannheim, Germany). The assay had a detection limit of 0.35 nmol/L, and the intra-assay coefficient of variation (CV) ranged from 2.6% to 5.6%. FSH and testosterone levels were also measured using the COBAS e411 analyzer, following the standard protocol provided with the commercially available kit from Roche Diagnostics. The free androgen index (FAI) was calculated using the formula: FAI = (total testosterone/SHBG) x 100 (23).

### 2.3 Data analysis

Data analysis was performed using the Statistical Package for Social Sciences (SPSS) version 22.0 (SPSS, Inc., Chicago, IL, USA). Descriptive statistics were used to report normal variables as mean and standard deviation (SD), while non-Gaussian variables were presented as median (25th and 75th) percentiles. Categorical variables were expressed as frequencies and percentages. Independent Student T test and Mann-Whitney U test was performed for mean and median difference between control and osteopenia group. Bivariate associations between normal variables were assessed using Pearson’s correlation, while Spearman’s correlation was employed for non-normal variables. P-value less then <0.05 consider significant statistically.

## 3 Results

**Table 1** presents the key characteristics of the study participants categorized by their respective groups. The study consisted of 103 men aged 60 years or older, with an average age of 60.3 ± 7.2 years. The majority of participants (82.4%) were over the age of 60. Approximately 46% of the men were diagnosed with osteopenia or low bone mass, and within this group, individuals had a significantly lower BMI compared to those in the normal group (p<0.001). Furthermore, the osteopenia group exhibited significantly lower BMD in the spine and femoral neck, as well as lower T-scores in the spine (p<0.001) in comparison to the normal group. In terms of hormone levels, the osteopenia group displayed significantly lower free androgen index (FAI) and higher levels of SHBG and FSH compared to the control group. However, there were no significant differences observed in age and testosterone levels between the two groups (**Table 1**). **Figure 1** provides a graphical representation of the circulating levels of SHBG and FAI in both study groups, based on our collected data.

**Table 1:** Clinical characteristic of the subjects.

Parameters	All	Control	Osteopenia	*p-value*
N	103	56	47	
Age (year)	60.3 ± 7.2	59.6 ± 6.9	61.3± 7.4	0.24
BMI (kg/m^2^)	29.8 ± 4.8	30.9 ± 4.6	28.4 ± 4.8	0.01
WHR	0.99 ± 0.1	0.99 ± 0.1	1.01 ± 0.1	0.10
T-Score (Spine)	-0.70 ± 1.3	0.28 ± 0.95	-1.86 ± 0.5	<0.001
T-Score Femur	-0.31 ± 1.2	0.17 ± 1.3	-0.89 ± 0.9	<0.001
Lumbar spine BMD (g/cm^2^)	0.97 ± 0.1	1.02 ± 0.1	0.91 ± 0.1	0.01
Femur neck BMD (g/cm^2^)	0.99 ± 0.2	1.0 ± 0.2	0.93 ± 0.1	0.005
SHBG (nmol/l)	36.2 (26–43)	31.1(25-39)	40.6 (29-51)	0.002
FSH (miU/ml)	4.4 (3.3-6.5)	4.0 (3.2-6.1)	5.2 (3.4-8.2)	0.07
Testosterone (ng/ml)	5.0 (4.1-6.9)	5.2 (3.7-6.9)	4.9 (4.1-6.7)	0.23
FAI (Testosterone/SHBG)	51.1 ± 15.5	55.1 ± 16.5	46.2 ± 12.8	<0.001

P-values are obtained from independent sample t-test, adjusted for age and BMI; p<0.05 considered significant. BMI, body mass index; FAI, free androgen index; SHBG, sex hormone binding globulin; WHR, Waist to Hip Ratio.

**Table 2** showed patient’s characteristic regarding comorbidities and risk factors. In the family history assessment, a significant prevalence of diabetes mellitus (DM) was observed in both the control (77.8%) and osteopenia groups (74.5%). However, dyslipidemia was notably higher in the osteopenia group at 12.8% compared to 1.9% in the control group (p=0.037). For medical history, diabetes mellitus (DM) was again prominent in both groups, with 79.6% in controls and 71.7% in osteopenia. A marked difference was seen in thyroid diseases, prevalent in 9.3% of the control group but absent in the osteopenia group (p=0.04). Other conditions showed comparable frequencies between the groups.

**Table 2:** the patient’s characteristic regarding comorbidities and risk factors.

Parameters	Control	Osteopenia	P-value
Family History			
DM Heart Disease Cancer HTN Dyslipidemia Osteoporosis Arthritis Disease Bone Fracture	42 (77.8) 6 (11.1) 0 (0.0) 22 (40.7) 1 (1.9) 7 (13.0) 1 (1.9) 1 (1.9)	35 (74.5) 4 (8.5) 1 (2.1) 14 (29.8) 6 (12.8) 8 (17.0) 0 (0.0) 3 (6.4)	0.44 0.46 0.46 0.17 0.037 0.38 0.47 0.26
Medical History			
DM Chronic Immobilization Cancer Thyroid Kidney Liver Rheumatoid Arthritis Vitamin D Deficient Inflammatory bowel disease Chronic Ulcer Cushing Grave Anorexia Bulimia Back Pain	43 (79.6) 6 (11.1) 5 (9.3) 5 (9.3) 2 (4.0) 0 (0.0) 2 (3.7) 4 (7.4) 0 (0.0) 0 (0.0) 0 (0.0) 4 (7.4) 0 (0.0) 0 (0.0) 3 (5.6)	33 (71.7) 1 (2.2) 1 (2.2) 0 (0.0) 3 (6.5) 0 (0.0) 1 (2.2) 4 (8.7) 0 (0.0) 0 (0.0) 0 (0.0) 1 (2.2) 1 (2.2) 1 (2.2) 3 (6.5)	0.25 0.09 0.14 0.04 0.46 — 0.56 0.55 — — — 0.24 0.46 0.46 0.58

Data presented N (%).P-value significant at 0.05 and 0.01 level using chi-square and fisher exact test. —; denotes no p-value.

### 3.1 Bivariate correlations of SHBG and FAI with other parameters

**Figure 2:**
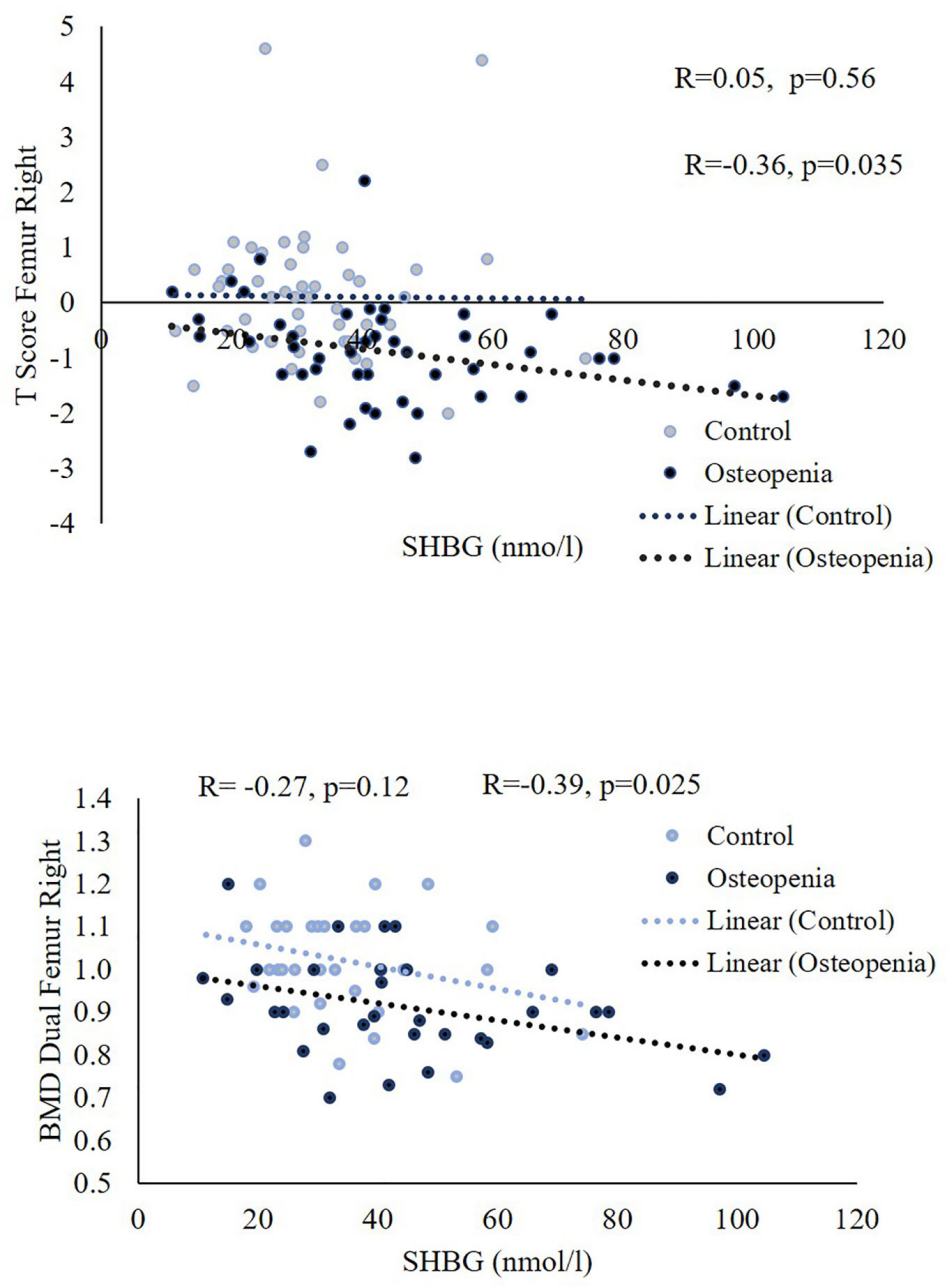
Scatterplots showing the associations of SHBG with spin BMD and T-score in the control and study groups.

The relationships between SHBG, age, testosterone, T-score, BMD spine, FAI, and BMI in different study groups are presented in **Table 3**. The findings demonstrate that SHBG levels were positively and significantly associated with age in both the control and osteopenia groups. Furthermore, there was a positive and significant correlation between SHBG levels and testosterone across all groups. However, in the osteopenia group, SHBG levels exhibited a significant inverse correlation with T-score femur, BMD spine, and FAI. Testosterone showed a significant positive correlation with age in the osteopenia group, while displaying an inverse correlation with BMI within the same group. No significant correlations were observed between testosterone and T-score (spine) or BMD spine in any of the study groups. Additionally, there were no significant correlations found between testosterone and FSH. On the other hand, the FAI demonstrated a positive and significant correlation with T-score femur in the osteopenia group and all subjects, while exhibiting an inverse correlation with FSH in the control group and all subjects. **Figure 2** provides a visual representation of the bivariate correlations between SHBG and T-score femur, as well as BMD (spine).

**Table 3:** Associations between SHBG, Testosterone and FAI with Anthropometric and BMD measures.

Parameters	SHBG			Testosterone			FAI		
	All	Control	Osteopenia	All	Control	Osteopenia	All	Control	Osteopenia
Age (year)	0.28**	0.18	0.33*	0.16	0.08	0.31^*^	-0.07	0.00	-0.06
BMI (kg/m^2^)	-0.07	0.07	-0.06	-0.24^*^	-0.21	-0.33^*^	-0.15	-0.25	-0.28
WHR	-0.06	-0.05	-0.22	-0.07	-0.02	-0.21	-0.04	0.00	0.04
T-Score Spine	-0.22*	0.04	-0.15	-0.08	-0.10	-0.16	0.24^*^	0.00	-0.01
T-Score Femur	-0.23*	0.05	-0.35*	-0.16	-0.26	-0.03	0.14	-0.20	0.36^*^
Lumbar spine BMD (g/cm^2^)	-0.38**	-0.27	-0.39*	-0.10	-0.02	-0.20	0.36^**^	0.35	0.26
Femur neck BMD (g/cm^2^)	-0.22	0.01	-0.30	0.00	0.08	-0.08	0.28^*^	0.17	0.32
FSH (miU/ml)	0.13	0.07	0.08	-0.11	-0.26	0.02	-0.22^*^	-0.28^*^	-0.11
Testosterone (ng/ml)	0.56**	0.60**	0.58**	1	1	1	0.45**	0.61**	0.22
FAI	-0.30**	-0.041	-0.48**	0.45**	0.61**	0.22	1	1	1
SHBG (nmol/l)	1	1	1	0.56**	0.64**	0.65**	-0.30**	-0.04	-0.48**

Data presented co-efficient of determination (R). * and ** presented p-value significant at 0.05 and 0.01 level. BMI, body mass index; FAI, free androgen index; SHBG, sex hormone binding globulin; WHR, Waist to Hip Ratio.

### 3.2 Stepwise regression analysis

To investigate the influence of SHBG, testosterone, and FAI on T-scores and BMDs, a stepwise linear regression analysis was performed. T-score and BMD were considered as the dependent variables, while SHBG, testosterone, and FAI served as the independent variables. The results of this analysis are presented in **Table 4**. The study findings indicated that among men with osteopenia, FAI exerted a significant impact on spine BMD (p = 0.024). However, SHBG did not demonstrate a significant effect on either T-scores or BMDs across any of the three groups.

**Table 4:** Stepwise regression analysis: T-score and BMD as dependent parameters.

Parameters	B (SE)	P-value
SHBG Testosterone FAI (Testosterone/SHBG)	T-Score Spine -0.06 ± 0.04 0.07 ± 0.07 -0.01 ± 0.03	0.12 0.30 0.68
SHBG Testosterone FAI (Testosterone/SHBG)	T-Score Femur -0.06 ± 0.03 0.08 ± 0.07 -0.02 ± 0.02	0.10 0.23 0.38
SHBG Testosterone FAI (Testosterone/SHBG)	BMD Femur 0.0001 ± 0.006 0.0001± 0.01 0.003 ± 0.004	0.94 0.98 0.48
SHBG Testosterone FAI (Testosterone/SHBG)	BMD spin 0.005 ± 0.004 -0.013 ± 0.008 0.007 ± 0.003	0.25 0.11 0.02

Data presented as beta-coefficient (β) and standard error (SE). p-value < 0.05 is considered significant.

## 4 Discussion

SHBG and testosterone are essential components of bone metabolism, but their link with BMD in older Saudi men hasn’t been studied much. This study examined the relationship between BMD and serum levels of SHBG, testosterone, and FAI in 103 middle-aged Saudi men. The study found that osteopenia patients had significantly increased SHBG and FSH levels, while FAI levels were reduced, which may explain bone loss. The study also found interesting associations between these markers and anthropometric indices. A notable observation in the osteopenia group was the significant increase in SHBG and testosterone levels with advancing age. These findings align with previous studies conducted in diverse populations, further supporting their consistency (24–26). Previous research has consistently demonstrated a significant decline in BMD after the age of 50, and this decline has been linked to variations in sex hormones (27, 28). There is a well-established reduction in sex hormone levels and functional hormone receptors, which are known to play a crucial role in the development of osteoporosis (13). Correlations of these markers with men age also propose a modulatory influence of age on elderly men sex hormones that then effect on bone metabolism. SHBG is a primary carrier for testosterone and higher levels of SHBG bind more free testosterone. So both SHBG and testosterone increases with age. These results indicate that SHBG and testosterone levels may be useful aging-related biomarkers in a group at increased risk of age-related diseases such physical function impairment and osteopenia.

Our research emphasizes the significance of recognizing obesity as a risk factor for osteopenia in men due to its impact on reducing serum testosterone levels. Testosterone is inversely associated with BMI in all and osteopenia groups. The relationship between obesity and low serum testosterone levels in men is still being debated (29), possibly due to the conversion of testosterone to estradiol by adipose tissue (30). This evidence of a cause-and-effect relationship between BMI and serum testosterone is backed up by a meta-analysis study of the effects of body weight loss on serum testosterone, which found that both a low-calorie diet and bariatric surgery are linked to a significant rise in serum testosterone after body weight loss (31). Osuna et al. (32) showed that as the BMI score goes up, the concentration of testosterone and SHBG goes down in the same way. Shamim et al. (33) did a study that was similar to ours. They found that as the BMI index went up, the amount of testosterone in healthy men ages 30–50 went down. Our study shows that this relationship is also true for men between the ages of 50 and 70.

This study revealed a notable inverse association between sex hormone-binding globulin (SHBG) and bone mineral density (BMD) at the spine. Conversely, testosterone levels exhibited a significant positive correlation with SHBG. As a result, it was anticipated that higher levels of serum testosterone would be linked to improved bone health. However, some studies reported a significant relationship between testosterone levels and BMD (26, 34). Additionally, low testosterone levels were associated with bone loss in other studies (13, 35, 36). In our study, we did not find any significant associations between testosterone levels and BMD or T-Score. These findings are consistent with previous research (37–39).

Recent studies have presented conflicting findings regarding the association between SHBG and testosterone with bone metabolism (39–41). For instance, the European Male Ageing Study (EMAS) revealed that testosterone was not linked to bone metabolism parameters in middle-aged and elderly men (39, 41). Interestingly, one study indicated that SHBG, rather than total testosterone, exhibited a significant relationship with biochemical bone turnover markers (BTMs) (39). In our own study, we observed a significant negative correlation between SHBG levels and both Femur T-Score and spine BMD. However, only FAI levels showed a significant positive correlation with T-Score Femur. These findings suggest that FAI levels, rather than testosterone levels, may be more closely associated with bone metabolism. A commonly acknowledged hypothesis regarding the role of SHBG in bone metabolism revolves around its anti-estrogenic effect. It is believed that elevated levels of SHBG bind to estrogen, thereby decreasing its biologically active form. This, in turn, can lead to a reduction in bone mineral density (BMD) and an increased susceptibility to fractures.

This research found no statistically significant relationship between SHBG and either BMD or t-scores when using multiple regression models. However, FAI was the only gonadal hormone that was a significant predictor of bone mass, and this relationship was only found to be significant at the spine. These results were the same as ones that had already been released (42). This could mean that androgens affect bone density in other ways, such as by affecting the function of the kidney’s 1a-hydroxylase, as suggested by Francis et al. (42) We used FAI as a measure of androgen state because it looks at both total testosterone and how much of it is bound to SHBG. Taxel et al. (43) found that when an aromatase inhibitor is given to older men, the levels of estradiol go down and bone resorption markers go up. Androgen receptors are present on osteoblasts (44), while both osteoblasts and osteoclasts have estrogen receptors (44, 45). Since osteoblasts make aromatase (46) it is likely that androgens are changed into estrogens at the tissue level.

One notable strength of this study is its novelty, as it is the first known investigation to examine the relationship between sex hormone-binding globulin (SHBG) and testosterone levels with BMD specifically in Arab middle-aged men. However, it is important to acknowledge the limitations of the study. The sample size was relatively small, which restricts the generalizability of the findings. Additionally, being a cross-sectional study, it only provides a snapshot of the proposed relationship, limiting our ability to establish cause and effect. Conducting a longitudinal study within this population would be valuable and informative in further exploring and understanding this relationship. Furthermore, it should be noted that we did not perform exclusions based on factors such as spondylarthrosis, which could potentially impact the accuracy of lumbar spine BMD measurements. This limitation is important to consider when interpreting our results and their relevance to the broader population of Arab middle-aged men.

In conclusion, the prevalence of osteopenia is related to greater SHBG concentrations and decreased FAI. In Saudi older men with osteopenia, SHBG was significantly inversely associated with BMD, notably at the spin. In addition, in the osteopenia group, FAI was positively and significantly correlated with femoral T-score. Age and menopause likely trigger a protective response of increased blood SHBG and testosterone levels in response to increased bone resorption. In comparison to BMD and DXA, that are direct measures of bone density, SHBG levels offer insights into the hormonal dynamics influencing bone health. As a diagnostic tool for osteoporosis or osteopenia, SHBG would act more as an adjunct or a supplementary test, rather than a replacement for BMD or DXA. More research is needed to understand the processes driving these correlations.

## Data availability statement

The original contributions presented in the study are included in the article/supplementary material. Further inquiries can be directed to the corresponding author.

## Ethics statement

The studies involving humans were approved by Ethics Committee of the College of Science, King Saud University, Riyadh, Kingdom of Saudi Arabia (Approval# 8/25/454266, 30 September 2013). The studies were conducted in accordance with the local legislation and institutional requirements. The participants provided their written informed consent to participate in this study.

## Author contributions

SY drafted the first version of the manuscript, MK made the statistical analysis. supervised critically revised the manuscript. ME contributed to the data collection. NA and AAcritically revised the manuscript. All authors contributed to the article and approved the submitted version.
